# Look for the meaning of rural school work: Qualitative study on identity of comprehensive teachers in rural primary schools in China

**DOI:** 10.3389/fpsyg.2022.1065906

**Published:** 2023-01-04

**Authors:** Yang Lian, He Xiaojun, Wu Cuiling

**Affiliations:** ^1^School of Teacher Education, Shaoguan University, Shaoguan, Guangdong, China; ^2^School of Education Science, Yulin Normal University, Yulin, Guangxi, China; ^3^Department of Education, Jeonbuk National University, Jeonju, South Korea

**Keywords:** rural schools, comprehensive teachers, identity, caring teachers, re-recognition

## Abstract

In the working environment of rural schools, whether and how does the identity of new comprehensive teachers change before and after service? Research on this problem is beneficial to optimize pre-service training and provide effective support for in-service training. In this study, phenomenological interviews were conducted to understand the formation and change of their identities in the three stages of pre-service, working and current teaching of four new teachers with 3-year teaching experience in the minority areas of western China. The results show that in the working environment of rural schools, three comprehensive teachers in primary schools have gradually changed from being excellent subject teachers to caring teachers. Another determined to become a caring teacher before her career, she retained her own identity in her work and focused more on the healthy growth of her students. The results show that the working environment of rural schools has a significant impact on the identity of first-term teachers of primary schools. Pre-service training should be integrated into local culture to promote the integration of pre-service and in-service and realize its sustainable development.

## Introduction

Rural teachers are the key to the development of rural education. At present, the core of rural teacher development is gradually transformed into the problem of identity. That’s because only when rural teachers identify themselves can they effectively improve the quality of education (Xian and Hua, 2019). In China, as an important source of rural teachers, new comprehensive teacher have been trained for more than a decade since 2006. However, the concept of “comprehensive primary school teachers” first started in the United Kingdom. They started from the requirements of comprehensiveness and integrity of students’ training, and carried out comprehensive training for teachers to cultivate their interdisciplinary teaching ability. However, the clear concept of “comprehensive primary school teachers” has not been put forward, and only uses some vague terms such as “comprehensive,” “interdisciplinary,” and “overall” (Wang, 2021). The clear concept of comprehensive primary school teachers was proposed by Chinese scholars after absorption and digestion. At present, there are still some differences on the definition of comprehensive primary school teachers in the academic circle, and a universally recognized and clear definition has not been formed. The comprehensive primary school teachers mentioned in this study refer to teachers who love education, have extensive knowledge, strong teaching ability, interdisciplinary thinking, and are good at interdisciplinary teaching, that is, teachers who have the ability to organically integrate primary school Chinese, mathematics, English and other disciplines with music, dance, art, calligraphy and other courses. The comprehensive teacher identity is affected by both personal and professional dimensions. Its identity characteristics are not only reflected in how teachers are viewed in interpersonal interaction, but also related to teachers’ self expectation and identification of their internal identity (Tilley, 2008). In rural schools with strong context and local characteristics, there are few studies on the rural education awareness, feelings and responsibilities of comprehensive teachers (Jiang, 2017; Tian and Xiong, 2020). In order to train excellent comprehensive teachers, the government has introduced a series of welfare policies, and many students with excellent conduct are attracted by this profession. Kang (2020) pointed out that the core of comprehensive teacher training is comprehensive development, applying knowledge to practical use, and learning with expertise. Achinstein et al. (2004) has proposed that during pre-service training, many comprehensive teachers are confident in their professional development. Because of the difference between the pre-service ideal and the post service reality of the special rural school environment (such as geographical location, weak teachers, short school construction time, poor cultural heritage, many left behind children, etc.), many new comprehensive teachers constantly reflect on the “significance of rural school work.” The study (Flores and Day, 2006; Niu, 2018) has found that early individual education practice and school work environment will have a significant impact on teachers’ identity.

In the working environment of rural schools, whether and what changes will happen to the pre service and post service identity of new comprehensive teachers. It is of great significance to optimize pre service comprehensive teacher training and provide support for post service comprehensive teacher professional growth. Based on the perspective of Social Identity Theory, this study uses phenomenological methods to investigate the process of the change of the identity of the four beginning teachers in the early rural school work environment and explore its essence and significance. Social identity theory is one of the most influential theories in the field of social psychology. It was proposed and developed by Tajfel and Turner in the 1970s. Its core view is that people define themselves by their social group qualifications, and the self perception of the social category to which individuals belong will have a unique psychological impact on their social behavior. The emergence of social identity has gone through three basic psychological processes: social classification, social comparison and positive differentiation (He and Wang, 2017). In this research, two problems will be studied: the first is what kind of initial identity the new comprehensive teachers have before their employment. Second, the impact of rural school environment on the new comprehensive teacher identity and its reasons.

### The meaning of teacher identity

The so-called “identity” is the basic answer to “who am I,” and “identity” is the individual teacher’s perception and judgment of the identity of teachers and their groups (Ling and Wu, 2019).

Identity is a teacher’s active pursuit of his own belonging, which includes both teachers’ individual understanding of “who I am” and others’ understanding of “who I am” (Buchanan, 2015). Lampert (2009) has pointed out that identity helps teachers understand themselves and their work, and has an impact on educational practice. Ruohotie-Lyhty (2013) has proposed that identity is regarded as a goal of teachers’ professional learning. Beijaard et al. (2004) has found that the teacher’s identity will change with the change of the working environment, and identity is a continuous process, rather than a stable one. Identity formation is the process of “explaining oneself as a certain person and being recognized as a certain person in a specific context.” Therefore, the examination of teachers’ identity in a specific context can be understood as the examination of “who am I now” (Day and Kington, 2008). The concept of how teachers view themselves and teaching can be regarded as identity.

Another problem that affects teachers’ identity is the relationship between identity and educational practice. It is generally believed that teachers’ identity cannot fully drive their own educational practice, but identity and educational practice are intertwined. Educational practice is formed by the interaction between teachers’ identity and various factors in their working environment (Ruohotie-Lyhty, 2013). When teachers explain and reinterpret their educational practice, their professional identity will be reconstructed and changed due to the mediation of their professional identity and working environment. In other words, identity and educational practice interact in a circular and iterative manner, rather than in a linear and causal manner. Analyzing the formation process of teachers’ identity is helpful to understand the meaning of teachers’ thinking and behavior, improve teachers’ educational practice and promote teachers’ professional development. Sachs (2001) specifically has pointed out that teacher identity can be understood as “teachers build their own frame work of how to be, how to act, and how to understand work and social status.”

### Studies on teachers’ identity

Previous studies have mainly focused on building a theory of teacher identity and understanding the nature and formation of professional identity by addressing the factors that affect teacher identity. Research has shown that factors related to teachers’ personal background (e.g., family background, life history, and pre-service education) and work environment (e.g., school culture, leadership, and student background) are critical to the formation of teacher identity (Ruohotie-Lyhty, 2013; Popper-Giveon and Shayshon, 2017). For the identity of beginning teachers, pre-service education experience and current school environment are two important factors that affect the formation and change of teacher identity. There are two distinct orientations of teacher identity: one is based on the subject knowledge and skills and their application in practice, to grow into excellent subject education experts. The other is growth as an “educator” that emphasizes the role of teachers in supporting students in their social, emotional, and moral development (Buchanan, 2015). It is found that teachers are more inclined to the identity of educator than the identity of subject education expert.

### Iteration of teacher identity based on school environment

Gatti and Catalano (2015) has found that school environment has a significant impact on teachers’ educational practice and professional learning. When the school environment conflicts with the teacher’s identity, the teacher’s identity will often change because of the school environment. That is, teachers tend to abandon original beliefs that conflict with the school environment and form identities that are appropriate to the school environment. The influence of school environment on the reconstruction of teacher identity is not a linear relationship, but a process in which teachers reconstruct their identity through actions and reflective actions based on the school environment. Through this process the initial identity of the teacher is iterated. That is, iteration is the process of forming a new teacher identity in action. Whereas the environment in which teachers work has a significant impact on shaping and changing their professional identity (Lasky, 2005). Most studies focus on ways to improve understanding of teacher professional identity and support teacher professional development. However, based on the special working environment of rural schools, the nature and formation of teacher identity are still ambiguous. Therefore, this study will explore how the identity of new comprehensive teachers changes at different stages and why it changes with the initial identity in the rural school work environment.

## Materials and methods

### Research design

In China, The Ministry of Education of China pointed out that the training goal of comprehensive teachers is to be excellent primary school teachers who love education and are competent for multidisciplinary education and teaching (Opinions of the Ministry of Education, 2014). Through the early professional learning, most comprehensive teachers in primary schools have constructed their own professional identity (Creswell, 2012). For example, grow up to be an “excellent teacher” or a “caring teacher.” How and why their identity changes after teaching is the main content of this study. The specific overview is in two aspects. First, what kind of initial identity does the primary school general teachers have before they take up their posts. Second, in the working environment of rural schools, whether and how their identity has changed. The research adopts the phenomenological research method, that is, to record the educational experiences of the research object, sort out and analyze them, obtain a deep understanding of the specific phenomenon, and explore the potential significance of the phenomenon in some aspects (Seidman, 2012).

### Research object

According to the research purpose, this study selected general subject teachers of primary schools who graduated from primary education major for 3 years in a local normal university in Guangxi, China. With their consent, four samples were selected by stratified sampling. The basic information of these four new comprehensive teachers in rural primary schools is as follows (Table 1).

**Table 1:** Basic information of comprehensive teachers.

Name	Gender	Qualification	Pre-service subjects	Teaching subjects	Years of teaching	Working place
Teacher A	Male	Undergraduate	Primary education (Mathematics)	Mathematics	3 years	Bobai County, Yulin City, Guangxi
Teacher B	Female	Undergraduate	Primary education (Mathematics)	Mathematics	3 years	Hechi City, Guangxi
Teacher C	Female	Undergraduate	Primary education (Chinese)	Chinese	3 years	Nanning, Guang Xi
Teacher D	Female	Undergraduate	Primary education (Chinese)	Chinese	3 years	Luchuan County, Yulin City, Guangxi

### Data collection

Phenomenological research requires long-term in-depth interviews to investigate the educational life experience of the research object. This research adopts the phenomenological interview method proposed by Seidman. This method consists of three interview stages, each of which has a different focus, covering the past, present and future of participants. According to Seidman’s guiding principles, the first interview focused on the education before becoming a teacher; The second interview obtained detailed information about the educational life of the new comprehensive teachers in the rural school working environment; The third interview required the new comprehensive primary school teachers to reflect on their pre service and current education life and look forward to their future career planning. Through three stages of interviews, we can understand the initial views of the research object’s educational experience, understand the nature and significance of the research object in constructing or reconstructing its educational experience, and avoid preconceptions and bias. All interviews were conducted at selected locations. According to Seidman, the average interview time is between 60 and 90 min. With the permission of the research object, the researcher made a recording and converted it into text. After the interview, the researcher also conducted an e-mail and telephone conversation to improve the information and clarify the questions raised in the interview. The research subjects received and checked the original texts of all interviews, confirming that the original meaning was retained.

### Data analysis

In order to understand the nature and significance of the identity of new comprehensive teachers in rural primary schools, the data analysis is conducted in two steps according to Moustakas, 1994 thematic analysis method. First of all, after fully understanding the educational experience of each new comprehensive primary school teacher, the research data was repeatedly read and highlighted, and their identity types were summarized and personal codes were assigned. For example, teacher B code is initial identity: proficient in mathematics, concerned about students, and concerned about academic burden. Then, compare the personal codes of the four teachers, check their similarities and differences, and create a code list for the above teachers, which reflects the potential nature of the research object. If a code is only related to one teacher, it is of great significance to the teacher and reflects his unique personal identity. Secondly, these codes are placed in three stages of the identification of comprehensive primary school teachers. On the basis of cross analysis, the essence and significance of the identification of new comprehensive primary school teachers are proposed, and further elaborated in the following research results and discussions. The following is the relevant code table (Table 2).

**Table 2:** The identity of the new comprehensive teachers in three different stages.

Research questions	Code	Teacher A	Teacher B	Teacher C	Teacher D
Professional identity of the new comprehensive primary teachers at pre-service stage	My parents supported me to become a rural primary school teacher	○	○	○	○
	I met an influential teacher	○	○	○	
	I studied hard to be admitted to the university	○	○	○	○
	I was nervous and stressed in high school	○	○	○	○
	I have always been a student with excellent character and learning	○	○	○	○
	I’m very interested in rural primary school teachers		○	○	
	I have accumulated rich educational experience in pre-service training				○
	I think there is a difference between pre-service learning and post-service teaching	○	○	○	○
	I want to teach my own course well	○	○	○	
	I think we should understand our subject and teach it well	○	○	○	
Professional identity of the new comprehensive teachers when working	I had difficulties at the beginning of my work	○	○	○	○
	I often work overtime	○	○	○	○
	I have a lot of administrative work in the school	○	○	○	○
	I was frustrated by exam oriented education	○	○	○	○
	I think students must prepare for exams if they want to get good grades	○	○	○	○
	I think it’s important to care about students	○	○	○	○
	I think the student’s learning experience is important	○	○	○	○
	I think it is easier to prepare lessons and attend classes than to manage students	○	○	○	○
	I think students should be encouraged and supported in their studies and life	○	○	○	○
Professional identity of the new comprehensive teachers at present stage	I hope to be an example to students		○		
	I want to know more about the students	○	○	○	○
	I think it is important to improve students’ interest in learning and sense of happiness	○	○	○	○
	I think autonomy in teaching is constrained	○	○	○	○
	I want to do better in attracting and supporting student development	○	○	○	○

## Research results and analysis

### The identities of the four new comprehensive primary school teachers in the pre-service stage: To become excellent subject teachers or caring teachers

During pre-service training, most new comprehensive primary school teachers formed their own initial ideas about what is good teaching and what kind of teachers to become, which constituted their initial teacher identity. According to the above data, except for teacher D, who wants to be a caring teacher, others want to be excellent subject teachers. Their identity formation process is analyzed as follows:

First of all, students have many common characteristics. They are all hardworking, have excellent academic performance, and have never violated discipline in school. Especially in high schools, they worked hard and achieved excellent results in the college entrance examination. Among them, teacher A and teacher B achieved outstanding results in mathematics in the college entrance examination, and teacher C and teacher D achieved outstanding results in Chinese. After the college entrance examination and the interview of teachers’ colleges, he was finally admitted by the primary school mathematics education major and the primary school Chinese education major.

Secondly, there are different motivations for choosing a teacher’s career. Teacher B and Teacher C said that their parents supported them in choosing a teaching career. In the process of growing up, he was deeply influenced by his favorite teachers and had a strong desire to become a teacher. In addition, selecting general teachers in primary schools can also obtain national policy support to reduce the family burden. Teacher A and Teacher D said that they did not have much interest in teachers, but they chose the major of general teacher education in primary schools. Teacher A said that he did not like or dislike the profession of teacher. Because his parents are teachers in rural schools, he chose the profession of teacher under the strong advice of his parents. Teacher D said that as a girl, teacher is a good job with stable work and good welfare. The primary school general teachers can not only obtain the economic support of the government, but also obtain employment security after graduation. It can be seen that there are both internal motivations and external work benefits to choose the profession of general teacher in primary schools, and various reasons are intertwined.

Thirdly, the influence of pre-service teacher education curriculum is different. Pre-service education focuses on learning curriculum knowledge. The content of the learning curriculum mainly emphasizes the theory and concept in the field of education and teaching. The education practice lacks the real teaching scene, especially the real environment of rural schools. As a condition of entry, in addition to the graduation certificate, they must obtain the teacher’s qualification certificate and Mandarin qualification certificate. Obtaining a teacher’s qualification certificate requires both written examination and interview. The written examination of the teacher’s qualification certificate belongs to the national examination. The examination content includes pedagogy, psychology, teachers’ laws and regulations, etc. The interview needs to complete lesson preparation within the time limit and pass the evaluation of the interviewer. This is a very challenging process. Obtaining the teacher qualification certificate shows that they have mastered solid basic knowledge of education and teaching and are competent for basic education and teaching tasks.

Among the four teachers, teacher A and teacher B have always had good math scores. They believe that learning math requires mastering solid basic knowledge, thinking more about problem solving methods, and aspire to be an excellent primary school math teacher. Teacher C loves literature and aspires to be an excellent primary school Chinese teacher. The common feature of these three teachers is that after pre-service teacher training, combined with their own life experience, they hope to grow into excellent subject teachers in the future. For example, teacher B said that I would like to be a math teacher liked by students in rural primary schools, to study math problems with students and improve their math scores.

Compared with the first three teachers, Teacher D should become a caring teacher. She said that she was not sure that she really liked teachers and was not sure that she was suitable for this profession. However, a course in educational philosophy completely changed her mind. In this course, he constantly reflected on “who I am” and “why I choose this major,” and finally found the meaning and motivation of becoming a teacher. She explained as follows: In reviewing her high school life, she realized how unhappy she was as a student. In addition to wanting to go to a good university, she had no plan for the future and no confidence. Therefore, as a teacher, we should help students with similar experiences to find the meaning and happiness of life. Just as my professor of educational philosophy has influenced me, I also want to be the kind of person who can touch the hearts of students.

### The identity of four comprehensive primary school teachers at the work stage: Adherence and re-recognition

After entering the rural school for the first time, the real scene of the rural school is gradually influencing the four teachers’ initial career pursuit. There are three main sources of these influencing factors: complicated school management tasks, a large number of left behind children’s education and their own education and teaching practices. These three aspects have brought great pressure to the new comprehensive teachers in primary schools.

First of all, they are required to play the role of educational administrators for the numerous and complicated school management tasks. Due to the shortage of teachers in rural schools, schools assign a lot of work and responsibilities to them. They not only have to play the role of teachers, but also play the role of school management. For example, teacher B said, “I thought I only needed to concentrate on teaching, but after I was assigned to primary school, I found that I needed to prepare too many materials for inspection, and there were often many dull forms to hand in. These tasks were boring, unnecessary, and time-consuming, but I had to complete them.” Teacher A said, “I have six roles to play in the school, namely, school information officer, student registration administrator, director of the teaching and research section, leader of the Young Pioneers, head teacher, and finally, the teacher. Because these management work takes up a lot of time, teaching becomes a” sideline. In reality, a large number of school management work takes up the time they can spend on curriculum planning and teaching. They are overwhelmed by these trivial jobs that have nothing to do with teaching, and these jobs are basically meaningless to them.

Secondly, the education of left behind children requires them to assume the dual role of teachers and parents. At present, there are many left behind children in rural schools, and they are lack of affection, so it is urgent to strengthen emotional education (Zhong and Zhu, 2017). As the important others of left behind children, rural teachers should not only “teach good books,” but also “educate good people,” assuming the dual role of teachers and parents. Teacher C said that there are 32 students in the third grade class I teach, including 10 parents at home, 11 parents at home, 9 living with grandparents, and 2 living with relatives. These left behind children are separated from their parents for a long time and lack of care. If they have problems, they can not be corrected in time. As time goes by, these left behind children have poor grades, poor hygiene, poor discipline, many bad habits, and serious school weariness. Teacher D said that his village primary school has established a management file for every left behind child, formed many mutual aid learning groups, and timely guided the students who found psychological problems to help them develop good learning habits and quality. In order to make the child more sunny, I set up a “Children’s Home” with the support of the school and carried out many activities. For example, opening a family phone, writing a letter to parents, holding regular calligraphy and painting exhibition competitions, and striving to be a “loving mother.”

Thirdly, teaching practice makes them constantly reflect on what kind of teachers they want to be. At present, quality education has been deeply rooted in the hearts of the people. Quality education should not only make students “learn well,” but also “study hard.” Therefore, teachers should not only have solid professional knowledge, but also have rich knowledge of educational psychology. Teachers should not only “impart knowledge,” but also “educate people.” In rural teaching practice, these four teachers have deep experience. Teacher A, Teacher B and Teacher C originally aspired to be excellent subject teachers, but in the real rural education practice, they gradually realized that teaching was not as expected. Teacher A said that he thought that students could learn mathematics only by explaining it clearly, but the actual effect was not ideal. Under the guidance of an old teacher, I gradually realized that teaching should not only teach knowledge, but also care about students’ needs, resulting in spiritual collision, and gradually realized the true meaning of “teacher orientation.” Teacher D said that in my class, I often observe students’ expressions because each expression contains rich content. I often encourage students to ask questions and debate with each other. When I saw the students’ progress in study, I felt heartfelt satisfaction.

It can be seen from the above that some changes have taken place in the real rural school education work of these four teachers. No matter their initial status is to become excellent subject teachers or caring teachers. The real working environment of rural school education really interferes with their pursuit of their initial identity and acts as an intermediary tool to rebuild their professional identity.

### The identity of the four new comprehensive primary school teachers at the current stage: Growing into caring teachers

Among the four teachers, teacher A, teacher B and teacher C have gradually grown into caring teachers from the original expectation of becoming excellent subject teachers. Teacher D retained her original identity, that is, to grow into a caring teacher. At the same time, they also said that they would provide more support for the healthy growth of students in the future. This shows their firm identification with their current identity. This change process is analyzed below:

First of all, the mainstream environment of rural schools is constantly promoting their transformation into caring teachers. First, in reality, teachers’ multiple identities are changing their understanding of teachers, and school management is also an important part of teachers’ work. For example, Teacher A said that because of the shortage of teachers, he has successively assumed six different roles, and school management has taken up a lot of his time, but this is also part of the teacher’s work. Second, caring for the healthy growth of students requires more patience and tolerance, especially the current outstanding education for left behind children, which requires teachers to pay more attention to the healthy development of students. Teacher B and Teacher C said that for the education of left behind children, the higher education administrative department has issued a series of policies. Caring for the healthy growth of children has become an important part of rural school education and the basic responsibility of rural teachers. Teacher D said that it is my basic duty to help many left behind children who are either emotionally weak or enjoy leisure rather than work or indulge in the Internet. Third, professional development is restricted to varying degrees. The four teachers all said that due to the influence of the school management system and teaching habits, the acquisition of teaching knowledge and the training of teaching skills mainly depend on the instructions of the higher education authorities. In daily teaching and research activities, “follow” and “imitation” are the most common forms of teachers’ professional development. Due to the remote geographical location of rural schools, scattered schools, and small number of teachers, there are few opportunities for research between schools and within schools. Occasionally in teaching research, due to the lack of scientific research methods, discourse expression is rarely recognized. These limits the professional development of new comprehensive teachers in primary schools to varying degrees, further promoting their continuous transformation to caring teachers.

Secondly, local feelings and educational practice constantly promote them to form caring teachers. First, the local feelings let them agree that developing rural education is their responsibility. All general subject teachers in primary schools are targeted to recruit students and obtain employment “from where they come from, back to where they go.” Most of them who were born and grew up in the countryside have strong local feelings and are willing to contribute more to rural education. Teacher A, whose parents are rural teachers, said that there are two or even three generations of students in the village who are my parents’ students. The teacher-student relationship blends with the neighborhood relationship, which makes me realize my obligation and responsibility to contribute to rural education. Second, the educational practice gradually made them realize that education should not only “preach, impart knowledge, and solve doubts,” but also “cultivate the mind, cultivate feelings, and become adults.” The four teachers all said that rural schools have their own special environment (for example, there are many left behind children), and learning to care is far more important than the subjects they teach. Teacher A said that it is more important to understand students’ lives and thoughts, establish close relationships with them, and cultivate their personalities than to simply teach knowledge. Teacher C said that she now insists on holding three parent meetings a semester to better understand students and establish close relationships with them. Teacher D said that she wanted to be a caring teacher before she took up her job. Now she finds that it is very valuable and meaningful for rural schools, but she is distressed by the lack of effective education methods. Through the practice of rural education, three teachers gradually transformed into caring teachers, and one teacher became more determined to become a caring teacher.

### Analysis of the reasons for the above identity change

The reasons of the above identity change of the new comprehensive teachers in primary schools as the followings:

For the four general subject teachers in primary schools, Teacher D retained his original caring teacher identity, and the other three teachers gradually grew from excellent subject teachers to caring teachers. That is, the four teachers have formed their current identity in the specific rural school working environment. Its current identity can be understood as the reconstruction of its professional identity in the working environment of rural schools according to the interpretation and reflection of educational behavior. That is, under the working environment of rural schools, the four caring teachers iterated their initial identities. The reasons are as follows:

#### The working environment of rural schools eliminates the initial identity of teachers

The specific performance is:

(1) The working environment of rural schools dispelled their initial teacher identity. The specific performance is as follows:

First, the management burden of rural schools dissipates their energy for professional promotion. The geographical location of rural schools is relatively remote, the level of regional economic development is relatively backward, and the educational resources are relatively scarce. With the rapid development of rural schools, due to the shortage of teachers, general subject teachers not only have to undertake a lot of administrative work, but also need to undertake a variety of tasks such as the education of left behind children, the control of dropout and school protection. These tasks take up a lot of time, which makes them feel that teaching has instead become a “sideline,” leading them to feel that the professional level of teachers has declined.

Second, the teaching concept of rural schools has hindered their professional ability. Compared with urban teachers, the teaching level of rural teachers lags behind that of urban teachers in terms of teaching philosophy and value orientation, teaching methods and the use and development of curriculum resources (Lu, 2013). On the one hand, the teaching in rural schools is still teacher led, and the teaching process of teachers’ teaching and students’ listening is repeated in the classroom. On the other hand, due to the limitations of the objective environment and the influence of utilitarianism, rural teachers often equate teaching effectiveness with students’ test scores. Programmed teaching process has become the first choice for teachers because of its advantages of short time and quick results.

Third, the lack of teaching and research ability in rural schools hinders the improvement of their professional ability. Teaching research is an important way to promote teachers’ professional growth. For a long time, the teaching, teaching and research of rural schools in China has been faced with such problems as decentralized layout, weak foundation, unclear objectives, and single training path (Lin, 2019). On the one hand, many rural teachers have not learned systematic education and teaching theories. Due to the relative lack of educational and teaching theories, the slow updating of knowledge, the lack of teaching and research experience, the lack of guidance in teaching skills and teaching methods, and the low efficiency of teaching, most rural teachers are in a confused and helpless state. On the other hand, rural teachers tend to attach more importance to teaching than research. They are not good at learning and accumulation at ordinary times. They do not often pay attention to the external educational trends. Education and teaching stay at the level of personal experience, and it is difficult to collectively discuss or solve the doubts and problems encountered in teaching from the theoretical and practical levels. In addition, some teachers do not have enough ideological understanding, think that teaching and research activities are optional, teaching and research can not be fully invested, and lack of due thinking. Although the education administration department has done a lot of work in the development of rural teachers, most of them are promoted by experience without in-depth thinking and systematic planning. In addition, most of the common rural teacher training is simple “sending classes to the countryside’, teacher salons and a series of special forums. Because they are divorced from the reality of rural teaching, the training content is difficult to meet the needs of teachers’ personalized development. All these hinder the provision of rural teachers’ teaching and research ability, and affect their professional ability.

(2) Many unique educational practices in rural areas have generated their caring teacher identity. The specific performance is as follows:

First, the characteristics of rural school students have changed their educational concepts. The core task of rural education is to promote the development of rural students, and the core is to cultivate the resilience of rural students (Xiong and Lin, 2020). At present, the resilience of rural students is mainly characterized by interaction, difference and integrity. As an important person who can obtain emotional support for rural students, rural teachers can guide students only on the basis of understanding them, and then give students confidence and strength to overcome difficulties and cultivate students’ resilience (Li et al., 2016). However, in the process of escaping from the countryside, rural teachers cannot devote themselves to teaching activities, understand and accept students, and create opportunities for students’ resilience. At the same time, rural teachers enter teaching activities as bystanders, and the concept they transmit to students is no longer to return home after learning, but to “become urban people.” They can not shoulder the responsibility of guarding and inheriting the local culture, nor can they help students establish deep feelings for the local, which dispels the students’ local complex.

Second, the special educational task of rural schools has changed their educational action. The low level of rural students’ learning management is caused by many reasons. On the one hand, because the students at the compulsory education stage are basically under age, their self-control is not strong, their self-control ability is relatively poor, and their self-study management ability is relatively weak; On the other hand, a large part of rural students are left behind children. These students have gradually developed some bad behavior habits due to their lack of parental guidance and supervision in family education for a long time. In school education, they have encountered some teachers’ laissez faire management attitude towards them, making these students “unattended” children in the countryside. All kinds of bad habits formed in such a growth environment were slowly brought into the classroom and daily learning life, resulting in a low level of overall learning management of rural students. We should clarify the responsibility of the school, strengthen the daily management of the school, and strengthen the sense of responsibility of teachers. Schools and teachers shoulder the important mission of “teaching and educating people.” Schools are the second home of students. Teachers should take the initiative to care for students and love them. Only in this way can schools radiate vitality and students can move from “heteronomy” to “self-discipline.”

Third, the education of rural left behind children has changed their educational methods. With the increase of migrant workers, there are a large number of left behind children in the countryside. Left behind children can not be directly nurtured because their parents are not around, which is easy to cause or induce children’s bad personality factors. These bad personality factors often show or directly lead to children’s behavior problems and learning disabilities (Tan, 2011). Strengthen the construction of campus culture and teacher culture, create a harmonious and upward cultural atmosphere, so as to give full play to the infiltration and infiltration function of culture, and enhance the sense of school belonging and learning happiness of rural students. Specifically, the school should establish a portfolio of left behind children, assign special personnel to take charge, implement the home visit system, visit the families of left behind children regularly, understand the living conditions of left behind children, and focus on all aspects of left behind boys; Strengthen legal training to enhance the legal awareness and self-protection awareness of left behind children; Regularly carry out team activities, enrich the spare time life of left behind children, and strengthen the communication between left behind children and others.

## Discussion and conclusion

The research shows that the identities of the four new comprehensive primary school teachers are still at the stage of “teaching in the countryside,” and the identity transformation from “teaching in the countryside” to “teaching for the countryside” has not been realized. In order to achieve the goal of “teaching for the countryside,” the identity of general subject teachers needs to highlight the educational nature, deepen the identity category of rural teachers, and explain the emotional identity, responsibility identity and behavior identity of rural teachers.

### Enhancing the feeling of devoting to local education: The root of the identity of general subject teachers in primary schools

Emotional identity refers to the positive feelings that primary school general practitioners choose to generate from teaching in rural areas, love rural education, like rural students, and accept rural environment, so as to consolidate their identity. In the pre service training, the general teachers are all targeted training, originating from the countryside and returning to the countryside. They lack a clear understanding of rural education, so it is necessary to cultivate their feelings for rural education and aspire to “teach for the countryside.”

First of all, pre service training should strengthen local education. Rural schools have a strong regional and local character, and this special working environment puts forward specific requirements for the role and identity of rural teachers (Zhao and Xie, 2020). The localization of rural teachers refers to the cultivation of local rural teachers with rural feelings, regional identity and identity. For the general subject teachers with targeted employment, the curriculum system and community activities of pre service training in colleges and universities should, as appropriate, incorporate local content such as rural changes, rural culture, local knowledge and local customs. In terms of curriculum implementation, local education content should be infiltrated, and special topics such as the development and utilization of rural education resources, local education, education and management of left behind children, and management of rural boarding schools should be set up, Systematically cultivate students’ comprehensive quality of teaching in rural areas, guide students to master the essentials of employment, reduce or ease the challenges or difficulties they face in the adaptation stage, and lay a professional foundation for identity. This will not only help students to have a better understanding of rural education and its strategic significance, but also help them to form their identity of rural teachers in many aspects, such as professional awareness, professional feelings and professional ability, so as to take precautions to resolve the crisis of rural teachers’ identity.

Secondly, general subject teachers should take the initiative to understand the local conditions. Rural teachers should make full use of local development advantages, adjust measures to local conditions, integrate advantages and turn them into educational resources, develop local curriculum and school-based curriculum, introduce local culture into the campus and classroom, guide rural children to understand the rural world, and fill the gap of rural youth spirit caused by the decline of rural culture. To create the cultural imagination space of rural education, teachers who truly understand the countryside and the situation of rural teenagers are needed (Liu, 2006). At the same time, we should reform the current teacher assessment mechanism, integrate rural culture into the evaluation content organically according to the local actual situation, investigate the rural teachers’ understanding of local culture, and investigate whether teachers can let students develop and love local culture at the same time. In this process, rural teachers should be insiders and participants of local culture, rather than “marginal people” and “outsiders.”

### Professional growth implanted with rural characteristics: Motivation of comprehensive teachers’ identity in primary school

Rural schools have their special local cultural characteristics, and the professional growth of rural teachers cannot be separated from this special environment. For a long time, the vision design of rural teachers’ professional development is based on the reference of urban teachers, and its content setting, path selection and evaluation system are separated from the actual professional development of rural teachers. Therefore, we must listen to the professional development demands of rural teachers, give full play to their initiative in the process of professional development, fully affirm their irreplaceable role as rural teachers, help rural teachers form the will and motivation for sustainable development, and strive to improve the endogenous motivation for rural teachers’ professional development (Cai et al., 2018). When building the vision of rural teachers’ professional development, we should fully consider the personal vision of rural teachers, the actual situation of rural education and the actual needs of rural teachers’ professional development, and strive to achieve the organic fit between the expected goal and the actual needs of rural teachers’ professional development.

(1) The professional growth of rural teachers should be based on “rural.” Circumstances and localization constitute the basic characteristics of the countryside. The countryside is an important field for rural teachers’ professional development (Li and Zhou, 2010). The basis of rural teachers’ professional growth should be based on the countryside, taking full account of the actual situation of the countryside, rural schools and the practical problems of rural teachers. The motive force of rural teachers’ professional growth is “about the countryside,” which serves the rural revitalization. Rural teachers themselves should strengthen the initiative consciousness and independent ability of professional growth, and strive to seek opportunities for professional growth; On the other hand, the government and education departments need to create conditions, create a strong cultural atmosphere, provide a platform and opportunities for rural teachers, and support and promote their professional growth in multiple ways and in an all-round way. The purpose of rural teachers’ professional growth is “for the countryside.” The professional growth of “for the countryside” is to better “serve the countryside” and enrich “rural feelings,” not to “escape from the countryside” or “despise the countryside.” Teachers should promote the life development of rural students, improve the quality of rural education, promote the prosperity of rural culture, and further promote rural revitalization as the fundamental goal of rural teachers’ professional growth (Liu, 2018).

(2) The professional growth of rural teachers should highlight the “local character.” The basic attribute of rural teachers’ professional growth lies in “rural,” and its professional characteristic, which is different from that of urban teachers, lies in “rural.” “Locality” is the main feature of Chinese traditional society (Fei, 1999). The existence of rural teachers is based on local culture, and the professional growth of rural teachers can not lose “local.” This requires:

First, the concept of rural teachers’ professional growth should be integrated into the local characteristics. On the basis of deeply understanding the nature of education, teachers should form a rational concept of understanding rural education and correctly understand rural education; Identify with the professionalism and uniqueness of rural teachers; Actively study the physical and mental development characteristics of rural students and the characteristics of their living environment, actively create various colorful activities with life significance, excavate local culture with educational value, and flexibly apply it to education and teaching.

Second, local cultural knowledge should be integrated into the professional growth of rural teachers. As a rural teacher, we should understand and be familiar with the development history, customs, social psychology, moral feelings, etc. of the countryside, so as to carry out “local education” for students. However, this is not the case. Some scholars once criticized the content of rural school education, saying that “rural schools rarely impart folk cultural knowledge, humanistic and historical experience and practical agricultural technology in rural society, and the content of education and teaching is seriously lacking in local flavor (Wu, 2017).

The third is to increase the professional ability of local curriculum development. The rural teachers’ professional growth is more important to have the awareness and ability of curriculum development. As a village teacher, we should realize that a village, everything in the village, students, teachers, etc. are “courses” (Ge, 2015). These “courses” are all originated from the countryside, and “teachers” are the “protagonists” in developing these courses. Therefore, rural teachers should be able to accumulate, be sensitive to discovery, and be good at studying local cultural resources, so as to develop local curriculum resources from the top to the bottom, and select the essence of local culture from the rich field of rural culture that suits the direction and goal of curriculum development. At the same time, they should organize these local essence cultures according to a certain logical organization to facilitate students’ learning.

This study believes that in order to realize the transformation of the status of comprehensive teachers in rural primary schools as “teaching for the rural,” effective practical paths should be found before and after their employment, and a talent training and development system integrating government, pre-teacher cultivation organizations, rural schools and teachers should be built.

First of all, the government and relevant education authorities play a leading role in building a cooperation platform between the government, pre-teacher cultivation organizations and rural schools, and create a good environment for comprehensive rural teachers in terms of salary structure, training system, professional title evaluation and employment.

Secondly, in combination with the reality of rural schools pre-teacher cultivation organizations (e.g., colleges and universities) should set up a curriculum system that suits the ability structure, design a management system that meets the needs of learning, and use modern educational technology to improve the quality of pre-teacher cultivation of comprehensive teachers.

Third, on-the-job comprehensive teacher’s later development should supported by the rural schools. They should create a democratic management atmosphere, advocate harmonious colleague relations, improve teaching conditions, develop a fair and reasonable evaluation system, and enhance the sense of belonging and happiness of comprehensive teachers.

Fourth, on-the-job comprehensive teachers should improve their professional ability, form optimistic, kind and inclusive character, and adapt to professional needs; Plan the later career development, deepen the role consciousness, and more fully and accurately understand their inner needs; Cultivate professional quality, form innovative learning attitude, and improve professional emotion and faith quality.

## Data availability statement

The original contributions presented in the study are included in the article/supplementary material, further inquiries can be directed to the corresponding author.

## Author contributions

YL was responsible for the design of the overall research plan, implementation of the research, collection of data, and writing of the report. HX was responsible for guiding the research, revising the research plan, and participating in the writing of the article. WC was responsible for data collection, implementation of research, and data processing. All authors contributed to the article and approved the submitted version.

## Funding

This study is the research result of the major project “Qualitative Research on the Professional Identity of Guangxi General Practitioners” (project number: 2022JD05) of Guangxi Education Science Key Research Base in 2022 under the “Fourteenth Five Year Plan” of Guangxi Education Science, the research project of the Cultural Construction and Social Governance Center in Guangxi Ethnic Areas in 2018: Research on the Construction of Campus Culture in Ethnic Areas from the Perspective of Cultural Symbiosis (project number: 2018YJJD0003), and the Research of the 2022 Shaoguan University Doctoral Talent Introduction Project “Research on the Characteristics and Formation Mechanism of the Resilience of Outstanding Primary School Teachers” (No. 9900064602).

## Conflict of interest

The authors declare that the research was conducted in the absence of any commercial or financial relationships that could be construed as a potential conflict of interest.

## Publisher’s note

All claims expressed in this article are solely those of the authors and do not necessarily represent those of their affiliated organizations, or those of the publisher, the editors and the reviewers. Any product that may be evaluated in this article, or claim that may be made by its manufacturer, is not guaranteed or endorsed by the publisher.
